# A *Burkholderia pseudomallei* Outer Membrane Vesicle Vaccine Provides Cross Protection against Inhalational Glanders in Mice and Non-Human Primates

**DOI:** 10.3390/vaccines5040049

**Published:** 2017-12-09

**Authors:** Sarah M. Baker, Christopher J. H. Davitt, Natalya Motyka, Nicole L. Kikendall, Kasi Russell-Lodrigue, Chad J. Roy, Lisa A. Morici

**Affiliations:** 1 Department of Microbiology and Immunology, Tulane University School of Medicine, New Orleans, LA 70112, USA; sbaker@tulane.edu (S.M.B.); cdavitt@tulane.edu (C.J.H.D.); nmotyka@tulane.edu (N.M.); nkikenda@tulane.edu (N.L.K.); croy@tulane.edu (C.J.R.); 2 Tulane National Primate Research Center, Covington, LA 70433, USA; kerussel@tulane.edu

**Keywords:** *Burkholderia mallei*, persistence, bacteria, infection, glanders, outer membrane vesicles, vaccine, biodefense

## Abstract

*Burkholderia mallei* is a Gram-negative, non-motile, facultative intracellular bacillus and the causative agent of glanders, a highly contagious zoonotic disease. *B. mallei* is naturally resistant to multiple antibiotics and there is concern for its potential use as a bioweapon, making the development of a vaccine against *B. mallei* of critical importance. We have previously demonstrated that immunization with multivalent outer membrane vesicles (OMV) derived from *B. pseudomallei* provide significant protection against pneumonic melioidosis. Given that many virulence determinants are highly conserved between the two species, we sought to determine if the *B. pseudomallei* OMV vaccine could cross-protect against *B. mallei*. We immunized C57Bl/6 mice and rhesus macaques with *B. pseudomallei* OMVs and subsequently challenged animals with aerosolized *B. mallei*. Immunization with *B. pseudomallei* OMVs significantly protected mice against *B. mallei* and the protection observed was comparable to that achieved with a live attenuated vaccine. OMV immunization induced the production of *B.mallei-*specific serum IgG and a mixed Th1/Th17 CD4 and CD8 T cell response in mice. Additionally, immunization of rhesus macaques with *B. pseudomallei* OMVs provided protection against glanders and induced *B.mallei*-specific serum IgG in non-human primates. These results demonstrate the ability of the multivalent OMV vaccine platform to elicit cross-protection against closely-related intracellular pathogens and to induce robust humoral and cellular immune responses against shared protective antigens.

## 1. Introduction

*Burkholderia mallei* is a Gram-negative, non-motile, facultative intracellular bacillus and the causative agent of glanders [1]. Glanders is a zoonotic disease primarily of solipeds, although it is highly contagious and can infect humans. Although glanders has been eradicated in the United States, Canada, and Western Europe, sporadic cases still occur in Eastern Europe, Asia, South America, North Africa, and the Middle East [2]. The disease may present an as acute, rapidly progressive, lethal infection or as an indolent, chronic infection lasting many years. Chronic disease may manifest as a sub-clinical infection or with clinical signs such as nasal discharge, enlarged lymph nodes, and cutaneous ulcerations [3]. Treatment of glanders is complicated as *B. mallei* is naturally resistant to multiple antibiotics and resides within the intracellular niche of mammalian host cells. There is no commercially available vaccine for human or animal use. *B. mallei* was used as a biological warfare agent in World War I and World War II and is classified as a Tier 1 overlap select agent by the Centers for Disease Control and Prevention and the United States Department of Agriculture due to its bioweapon potential [4].

*B. pseudomallei*, which causes the disease melioidosis, is closely-related to *B. mallei* and is also listed as a Tier 1 select agent. *B. mallei* evolved from *B. pseudomallei* through genome reduction [5]. As such, many virulence determinants including surface polysaccharides, outer membrane proteins, secretion systems, and motility proteins are highly conserved between the two species [6,7,8,9] This suggests that it may be feasible to target both pathogens with a single vaccine platform composed of shared and conserved antigens.

Immunization with live attenuated vaccine strains has generated some of the best protection to date against both melioidosis and glanders in mice [10,11,12,13,14,15,16]. This is likely due to the multivalent nature of live attenuated vaccines and an ability to induce both humoral and cellular immune responses which are essential for full protection [17,18]. Live attenuated vaccines are attractive in this regard, but present several drawbacks including risk of reversion to virulence, horizontal gene transfer, and recombination with other bacteria. Furthermore, the use of live attenuated vaccine strains produced from highly virulent bacteria such as *B. pseudomallei* and *B. mallei* raise safety concerns for live vaccine applications. A number of purified subunit antigen preparations such as lipopolysaccharide (LPS) and capsular polysaccharide (CPS) and recombinant proteins have been evaluated and provide variable degrees of protection in small animal models [19,20,21]. Given the complexity of *Burkholderia* strains and their inherent genetic plasticity, it is considered unlikely that a monovalent subunit vaccine would be capable of generating sterilizing, broad spectrum protection against many different strains [19,21,22,23]. Additionally, the complex intracellular lifestyles of *B. pseudomallei* and *B. mallei* may necessitate a multivalent vaccine formulation that can induce protective immunity against multiple antigens expressed at different stages of infection [24]. In support of this, immunization with a mixture of subunit proteins or glycoconjugate formulations elicits significant protection against melioidosis in mice [24,25]. Furthermore, biological and synthetic nanoparticle formulations that incorporate more than one *Burkholderia* subunit antigen have shown promising results in both rodent and nonhuman primate model of melioidosis and glanders [26,27,28,29,30,31].

We were the first to demonstrate that immunization with multivalent outer membrane vesicles (OMV) derived from *B. pseudomallei* provided significant protection against pneumonic melioidosis in mice [29]. OMVs are non-infectious nanoparticles that are naturally secreted from the Gram-negative bacterial cell surface. OMVs incorporate multiple protective surface antigens, including proteins, lipids, and carbohydrates, which retain their native orientation and structure [32]. We have previously shown that OMVs confer significant protection against challenge with a heterologous *B. pseudomallei* strain [28]. This led us to hypothesize that the multivalent nature of the OMV vaccine platform could confer cross-protection against challenge with *B. mallei*.

In this study we evaluated the protective efficacy of a multivalent *B. pseudomallei* OMV vaccine against aerosol infection with *B. mallei* strain China 7 in mice and non-human primates. We also compared the immunogenicity and protective efficacy of the OMV vaccine with a live attenuated strain in the first head-to-head experimental challenge. We demonstrate that immunization of mice with the *B. pseudomallei* OMV vaccine provides significant protection against an otherwise lethal glanders infection caused by aerosol challenge. Furthermore, we show that OMV vaccine immunogenicity is superior to that induced by a live attenuated vaccine. Finally, we show that OMV immunization protects against disseminated glanders disease in nonhuman primates. 

## 2. Materials and Methods 

### 2.1. Ethics Statement

This study was performed in strict accordance with the *Guide for the Care and Use of Laboratory Animals* of the National Institutes of Health (NIH). The protocols were approved by the Tulane University Institutional Animal Care and Use Committee (P0276). The Tulane National Primate Research Center (TNPRC) is fully accredited by the Association for the Assessment and Accreditation of Laboratory Animal Care-International. Prior to challenge, mice were transferred to an Animal Biosafety Level (ABSL)-3 facility at the TNPRC and allowed to acclimate for one week prior to challenge. For survival studies, death was not used as an endpoint. Mice were humanely euthanized once they displayed >20% weight loss, paralysis, or were unresponsive to handling. Mice were observed at least three times daily, including weekends. Euthanasia was performed in mice by CO_2_ overdose and confirmed by cervical dislocation. Rhesus macaques were maintained in ABSL-3 housing. Remote biotelemetry was used to monitor nonhuman primates and began two weeks prior to infection to establish baseline parameters. Macaques were observed at least three times daily during the acute stage of infection. Euthanasia of macaques was performed at the study end point using an overdose of pentobarbital under anesthesia.

### 2.2. Bacterial Strains and Growth Conditions

*B. mallei* strain China 7 was obtained from BEI Resources. *B. pseudomallei* Bp82 was kindly provided by Herbert Schweizer and is a ΔpurM derivative of *B. pseudomallei* 1026 [33]. Bacteria were cultured from glycerol stocks immediately prior to use and single colonies were selected from freshly streaked LB agar plates. For live vaccination with Bp82, overnight cultures were diluted 1:100 in fresh LB supplemented with 100 μg/mL adenine hydrochloride (MilliporeSigma, St. Louis, MO, USA) and 5 μg/mL thiamine hydrochloride (MilliporeSigma) and incubated with shaking at 37 °C until the OD_600_ reached 1.0. For the challenge experiments, overnight cultures of *B. mallei* were diluted 1:100 in fresh LB with 4% glycerol (MilliporeSigma) and incubated with shaking at 37 °C until OD_600_ reached 0.75. 

### 2.3. OMV Purification

OMVs were purified as previously described [29,34,35] with minor modifications. *B. pseudomallei* strain Bp82 was freshly streaked from a glycerol stock onto LB agar and incubated for 48–72 h at 37 °C. An individual colony was inoculated into Luria broth (LB) and incubated at 37 °C for 16–18 h. The overnight culture was diluted 1:100 into fresh media supplemented with 100 μg/mL adenine hydrochloride (MilliporeSigma) and 5 μg/mL thiamine hydrochloride (MilliporeSigma) [33] and incubated at 37 °C for 16–18 h until late log phase (OD_600_ 4.5–5.0). Intact bacteria were pelleted by centrifugation (6000× *g* for 10 min at 4 °C) using an SLA-1500 fixed angle rotor. Following centrifugation the supernatant was filtered twice through a 0.22 μm polyethersulfone (PES) membrane (MilliporeSigma) to remove any remaining bacteria or large bacterial fragments. Absence of bacterial contamination was verified by incubating 2 × 0.5 mL of supernatant on LB agar for 48–72 h at 37 °C. OMVs were precipitated by incubating with 1.5 M ammonium sulfate (Fisher Scientific, Pittsburgh, PA, USA) overnight and then harvested by centrifugation (11,000× g, 20 min, 4 °C) using an SLA-1500 rotor. Crude vesicles were resuspended in 60% sucrose (MilliporeSigma) in 10 mM Tris-HCL pH 7.4, layered at the bottom of 35–60% density gradient, and subjected to ultracentrifugation (200,000× *g*, 3 h, 4 °C) using a 50.2Ti rotor. Fractions of equal volume were removed from the top, individually subjected to TCA precipitation, then evaluated by SDS-PAGE to visualize protein profiles by Coomassie blue staining as previously described [29]. Fractions containing identical protein profiles were pooled and subjected to ultracentrifugation (200,000× *g*, 1.5 h, 4 °C) to obtain highly purified vesicles (Figure S1). Purified vesicles were re-suspended in LPS-free water, visually confirmed by transmission electron microscopy, and quantitated by Bradford assay, as previously described [29]. 

### 2.4. Active Immunization and Challenge of Mice

Male and female C57Bl/6 mice, 8 to 10 weeks old, were purchased from Charles River Laboratories (Wilmington, MA, USA) and maintained 5 per cage in polystyrene microisolator units under pathogen-free conditions. Animals were fed sterile rodent chow and water *ad libitum* and allowed to acclimate 1 week prior to use. 

Mice (*n* = 15 per group) were immunized subcutaneously with 10^6^ colony-forming units (cfu) of live Bp82 bacteria or 10 μg of Bp82-derived OMVs diluted in 100 μL of sterile saline on day 0. Control animals were sham immunized with saline. Mice were given a booster dose three weeks after the initial dose. A subset of immunized mice (*n* = 5 per group) were utilized to evaluate antibody and cellular immune responses to vaccination and were not challenged. One month after the last immunization, immunized and control mice (*n* = 10 per group) were challenged by small particle aerosol as previously described [36] using a target dose of 1000 cfu of *B. mallei* strain China 7 (BEI Resources, Manassas, VA, USA). Actual mean infectious dose delivered to the mice was determined by plating the inoculum and all glass impinger (AGI) collections and was calculated to be 1246 cfu per mouse, which is an LD_50_ equivalency of 1.4LD_50_ [16]. Survival was monitored up to 30 days post-infection. Spleens were harvested from mice that survived to the study endpoint in order to assess persistent bacterial infection. Tissues were aseptically removed from euthanized animals, individually placed in 1 mL 0.9% NaCl, and homogenized with sterile, disposable tissue grinders (Fisher Scientific). Ten-fold serial dilutions of spleen homogenates were plated on LBG agar. Colonies were counted after incubation for 3 days at 37 °C and reported as cfu per spleen. The limit of detection was 10 cfu. Immunization and challenge experiments in mice were performed twice to confirm reproducibility.

### 2.5. Active Immunization and Challenge of Rhesus Macaques

#### 2.5.1. Immunization and Challenge 

Ten male Indian rhesus macaques (*Macaca mulatta*) were used in this study. Six animals (KM81, KI62, KH26, KL67, KP63, KT28) were immunized subcutaneously (SC) with 100 μg Bp82-derived OMVs. Four animals (KN25, NR40, KP06, KL22) were immunized SC with saline only (sham) and served as controls. Immunizations were performed on days 0 and 28. Blood was obtained prior to immunization (pre-immune), one month after the first immunization (prime), and two weeks after the second immunization (boost) for measurement of humoral and cellular immune responses to vaccination.

Four weeks after the final immunization, immunized and control macaques were challenged with a target dose of 10^6^ cfu (100LD_50_ equivalency [26]) of *B. mallei* strain China 7 by small particle aerosol using a head-only configuration as we previously described [37]. Actual infectious doses delivered to the macaques were determined by plating the inoculum and AGI collections and are presented in Figure S3.

#### 2.5.2. Monitoring of Respiratory Function 

The respiratory function of the macaques were measured by subjecting each animal to whole-body plethsymography at various time points throughout this study, including pre-challenge (naïve) measurement. Animals were anesthetized using tiletamine/zolazepam (Telazol, 6–8 mg/kg) and then placed in dorsal recumbency into a custom, sealed acrylic whole body chamber fitted with a 3 mm rubber dam surrounding the neck. The animal was allowed to breathe normally, and thoracic movement produced volumetric displacement of air in the sealed chamber measured by a pneumotachograph. Digital signal from passive respiratory maneuvers were acquired and converted to wave form for further analysis using specialized software (IOX2, SciReq, Montreal, QC, Canada). Respiratory function measurements were collected continuously for three minutes for a minimum of 18 sampling points per animal per sampling event. 

#### 2.5.3. Necropsy and Gross Pathology 

Three weeks after *B. mallei* challenge, all macaques were anesthetized (Telazol, 6–8 mg/kg) and given an opioid analgesic (Buprenex, 0.01 mg/kg) then euthanized with an overdose of pentobarbital. Necropsy and gross examination were performed by veterinary pathologists, and tissues were collected for subsequent analysis. Splenic weights were measured for comparison between groups.

### 2.6. Assessment of Antibody Responses to Vaccination 

Serum was collected from mice one month after the final immunization to evaluate antigen-specific antibody responses. *B. mallei*- and OMV-specific IgG serum antibody titers were measured by enzyme-linked immunosorbent assay (ELISA) using microtiter plates coated with OMVs or heat-killed *Burkholderia mallei* at a concentration of 500 ng/well in coating buffer and incubated overnight at 4 °C as previously described [29]. For murine ELISAs, additional wells were coated with purified mouse IgG1 to generate a standard curve (Sigma). Plates were incubated with two-fold serial dilutions of sera samples for 1 h at room temperature. Alkaline phosphatase (AP)-conjugated goat anti-mouse IgG secondary antibodies (1:300 dilution, Sigma) were added and incubated for 1 h at room temperature then developed and read as previously described [29]. Results were expressed as ELISA units/mL (EU/mL) using an average of sample dilutions closest to the midpoint of the standard curve. 

The presence of antibodies to *B. mallei*), and Bp82 OMVs was evaluated by Western blot. Ten μg of heat-inactivated B. mallei and Bp82 OMVs were separated by sodium dodecyl sulphate polyacrylamide gel electrophoresis (SDS-PAGE) using a 4.0–20.0% polyacrylamide gel (Bio-Rad, Hercules, CA, USA). The proteins were transferred to a nitrocellulose membrane and blocked in 1.5% BSA in TBS-T for 1 h. The membrane was incubated overnight at 4 °C with pooled sera (1:200 dilution) collected from mice (*n* = 5 per group) immunized with saline or Bp82 OMVs. The next day, the membrane was washed 3 times with TBS-T and incubated with goat anti-mouse IgG (1:5000, Thermo Fisher, Waltham, MA, USA) for 1 h at room temperature. The membrane was washed and developed using Opti-4CN substrate according to manufacturer instructions (BioRad). Blood was collected from rhesus macaques prior to the first immunization (pre-immune), one month after the first immunization (prime), and one month after the final immunization (boost) to evaluate antigen-specific antibody responses in sera. High-binding microtiter plates (Greiner Bio-One, Monroe, NC, USA) were coated as above and incubated at 4 °C overnight. All subsequent incubations were done at room temperature for two hours on an orbital shaker at 300 rpm. Coated plates were washed three times with wash buffer (1× PBS with 0.1% Tween 20). Washed plates were incubated with blocking buffer (5% skim milk in wash buffer) for 2 h. Plates were then washed three times. Washed plates were incubated with NHP serum serially diluted in blocking buffer from 1:4000 to 1:256,000. Plates were washed three times and then incubated for 1 h with goat anti-monkey IgG secondary antibody (Fitzgerald, Acton, MA, USA) diluted 1:500 in blocking buffer. Plates were washed three times and developed using 3,3′,5,5′-Tetramethylbenzidine (SeraCare, Milford, MA, USA). Color development was stopped using 1.0 M H_2_SO_4_. Plates were immediately read at 450 nm. Results for each animal were plotted as reciprocal titers versus absorbance, and endpoint titer was defined as the greatest dilution that yielded an optical density (OD_450_) greater than three standard deviations above the mean OD_450_ for pre-immune titers.

### 2.7. Assessment of T Cell Responses to Vaccination 

Murine T cell responses to immunization were assessed in spleens collected after the final immunization. Single cell suspensions were prepared by homogenizing organs on a 70 μm nylon cell strainer (Fisher) with a rubber syringe plunger from a 5 mL syringe (Fisher). The cell suspension was centrifuged at 460× *g* for 10 min at 4 °C. Supernatant was decanted and the cells were resuspended in 2 mL ACK red blood cell lysis buffer (Invitrogen, Waltham, MA, USA) and incubated at room temperature for 3 min and the reaction stopped with 20 mL of RMPI (Gibco, Waltham, MA, USA) containing 10% fetal bovine serum (FBS, Atlanta Biologicals, GA, USA), hereafter referred to as 10% RPMI. Cells were then centrifuged at 300× *g* for 10 min, supernatant was decanted, and the cells were resuspended in 5 mL 10% RPMI. The viable cells were counted on a Cellometer (Nexcelom Bioscience) using Trypan Blue (Sigma) and corrected to a final volume of 1 × 10^7^ cells/mL from which 1 × 10^6^ cells were added per well to a 96 well round bottom plate. Cells were stimulated with 0.4 μg purified anti-CD28 (eBioscience, Waltham, MA, USA) alone or together with 2.5 μg of OMVs or with 10ng PMA (Sigma-Aldrich) and 100 ng Ionomycin (Sigma-Aldrich) for 2 h at 37 °C with 5% CO_2_. GolgiPlug (BD Biosciences, San Jose, CA, USA) was added to cells to immobilize intracellular cytokines and the incubation was allowed to proceed for a further 6 h. Cells were washed once by centrifugation at 400× *g* for 3 min with 1× PBS. Cells were stained for viability using 0.1 μL Fixable Viability Dye-eFluor780 (eBioscience) for 30 min on ice. Following another wash step with FACS Buffer (2% FCS, 1 mM EDTA, 0.1% Sodium Azide in 1× PBS) cells were incubated with 0.1 μL anti-CD16/32 FcBlock (eBioscience) in FACS Buffer for 10 min on ice. A volume of 0.25 μg of the following stains for surface markers corresponding to CD3-BV605 (BD Biosciences; 1702), CD4-BV510 (BD Biosciences; RM4-5), CD8-PE-Cy7 (eBioscience; 5.3–6.7), CD44-eF450 (eBioscience; IM7) and dump markers redFluor710 (Tonbo Biosciences, San Diego, CA, USA; B220-RA3-6B2, CD11b-M1/70, CD11c-N418, CD19-1D3, F4/80-BM8.1) were added in FACS Buffer supplemented with brilliant violet staining buffer (BD Biosciences) for 30 min on ice. Cells were washed two times with FACS Buffer and incubated with Fixation/Permeabilization Buffer (BD Biosciences) for 60 min on ice. Cells were washed two times with Wash/Perm Buffer (BD Biosciences) and stains corresponding to CD3-BV605, IL-17A-PerCP-Cy5.5 (eBioscience; eBio17B7) and IFN-γ-PE (eBioscience; XMG1.2) overnight at 4 °C. Cells were washed twice in Wash/Perm and once in FACS Buffer. Samples were acquired on an LSR Fortessa (BD Biosciences) using FACsDiva (BD Biosciences) software. Data files were analyzed using FlowJo (Treestar, Ashland, OR, USA).

### 2.8. Statistical Analyses 

Statistical analyses were performed using GraphPad Prism version 5.0 (GraphPad Software, San Diego, CA, USA). *p*-values < 0.05 were considered statistically significant. 

## 3. Results

### 3.1. Immunization with B. pseudomallei OMVs Protects Mice against B. mallei 

**Figure 1:**
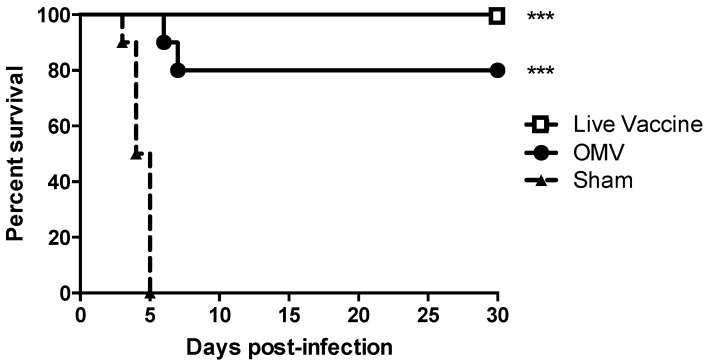
Immunization with *B. pseudomallei* outer membrane vesicles (OMVs) protects mice against pneumonic glanders. Mice (*n* = 10 per group) were challenged with 1246 cfu of *B. mallei* by small particle aerosol. Survival was monitored for up to 30 days. Mice immunized with OMV or live vaccine were significantly protected (*** *p* < 0.001 as determined by Log Rank Mantel–Cox test).

We tested the capacity of OMVs derived from the select-agent exempt, attenuated *B. pseudomallei* strain Bp82 [33] to cross protect mice against aerosol challenge with *B. mallei*. For these studies, we chose to use C57Bl/6 mice because they are better at mounting Th1-type immune responses compared to BALB/c mice [38]. C57Bl/6 mice were immunized subcutaneously with 10 μg Bp82 OMVs or 5 × 10^6^ CFU live Bp82 bacteria that has been shown to provide excellent vaccine protection against melioidosis in mice [12]. Control mice were sham immunized with saline only. All sham immunized mice succumbed to infection within 5 days of aerosol challenge. In contrast, immunization with OMVs significantly protected mice against an otherwise lethal aerosol challenge with 80% of mice surviving to the 30 day study endpoint (*p* < 0.001) (Figure 1). Immunization with live Bp82 also provided significant protection against aerosol infection with 100% of mice surviving to day 30 (*p* < 0.001). There was no significant difference in survival between OMV- and live Bp82 immunized mice (*p* = 0.15). These results indicate that the acellular *B. pseudomallei* OMV vaccine provides significant cross protection against *B. mallei* and induces protection comparable to a live attenuated vaccine. 

**Figure 2:**
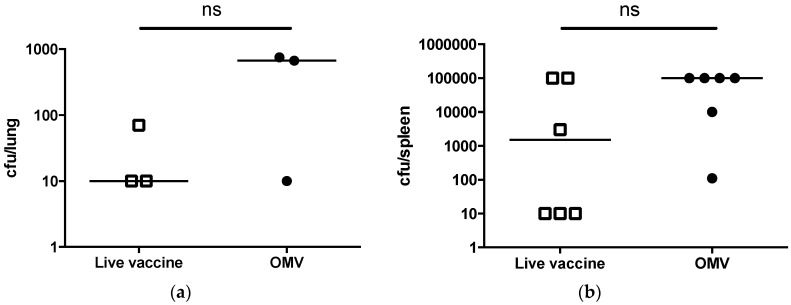
Immunized C57Bl/6 mice are persistently colonized with *B. mallei*. *B. mallei* bacterial burdens in the (**a**) lungs (*n* = 3 per group) or (**b**) spleen (*n* = 6 per group) of surviving mice immunized with OMV or live vaccine (ns = not significant; lungs, *p* = 0.13 and spleens, *p* = 0.09 by *t*-test).

All surviving animals were euthanized at the 30-day study endpoint and a subset of mice in each group were randomly selected for determination of bacterial persistence in the lungs and spleen. It was evident that *B. mallei* evaded complete immune clearance, leading to colonization and persistence in the lungs and spleen of live Bp82-immunized and OMV-immunized mice. Both live Bp82- and OMV-immunized mice displayed low levels of colonization (<750 cfu) in the lung (Figure 2a). Bacterial persistence in the lung may be a result of persistent colonization from the initial aerosol challenge or reseeding of the lung through hematogenous spread over the 30-day period. Mice were also colonized in the spleen with greater than 10^3^ CFU (Figure 2b), indicating that bacterial dissemination did occur in immunized animals. 

### 3.2. Immunization with B. pseudomallei OMVs Induces B. mallei-Specific Antibody in Mice 

**Figure 3:**
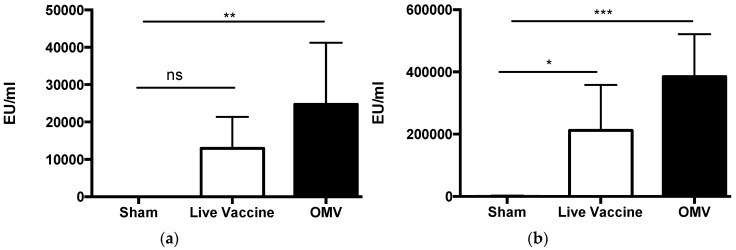
OMV immunization induces *B. mallei*-specific antibody in mice. (**a**) *B. mallei-*specific and (**b**) OMV-specific IgG was measured in the sera of mice (*n* = 5 per group) immunized with sham, live Bp82, or Bp82 OMVs by ELISA (* *p* < 0.05 ** *p* < 0.01 *** *p* < 0.001 by one way ANOVA, ns = not significant).

We previously demonstrated that *B. pseudomallei*-derived OMVs contain lipopolysaccharide (LPS), capsular polysaccharide (CPS) and numerous protein antigens [29], and that murine and nonhuman primate antibody responses generated to the OMV vaccine target both polysaccharide and protein components [28,30] Many of these components have been shown to be highly conserved in *B. mallei* [39,40]. For the current study, we postulated that immunization with *B. pseudomallei* OMVs would induce the production of *B. mallei*-specific antibody due to the presence of conserved surface antigens. We therefore measured the *B. mallei*-specific and OMV-specific IgG in the serum of mice immunized with OMVs, live vaccine, or saline only (sham). Immunization with OMVs induced the production of significantly more *B. mallei*-specific serum IgG compared to sham-immunized mice (*p* < 0.01; Figure 3a), confirming the presence of shared or cross-reactive antigens in the OMVs. This was confirmed by Western blot of whole *B. mallei* lysate (Figure S2). Immunization with live Bp82 did not induce the production of significantly more *B. mallei*-specific serum IgG compared to sham-immunized mice (*p* = 0.17) (Figure 3a). Immunization with both live Bp82 and OMVs induced a significant increase in OMV-specific serum IgG (*p* < 0.05 and *p* < 0.001, respectively) compared to sham-immunized mice (Figure 3b). Collectively, these data demonstrate that immunization with *B. pseudomallei* OMVs induces high titers of *B. mallei*- and OMV-specific serum IgG in mice.

### 3.3. Immunization with OMVs Induces Cellular Immune Responses in Mice

**Figure 4:**
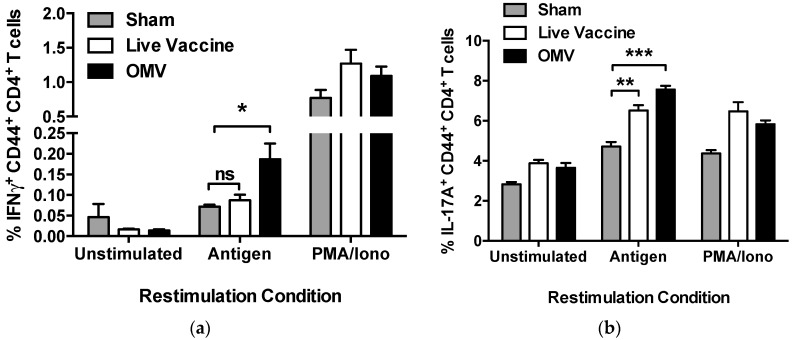

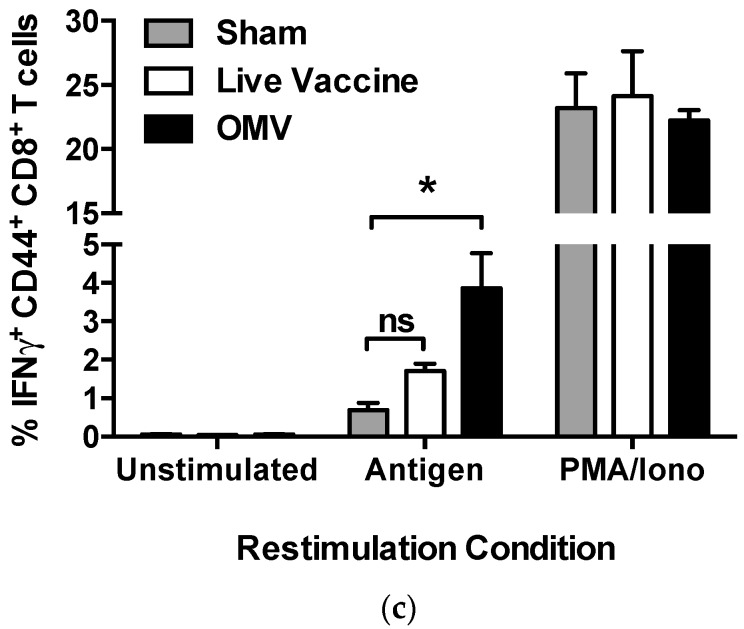
OMV immunization induces antigen-specific mixed Th1/Th17 CD4 T cells and CD8 T cells in mice. Antigen-specific CD4 and CD8 T cell responses were measured in the spleens of mice (*n* = 3 per group) immunized with sham, live vaccine, or OMV by flow cytometry (* *p* < 0.05 ** *p* < 0.01 *** *p* < 0.001 by one way ANOVA, ns = not significant). Cells were gated using granularity to include lymphocytes, then size to include only single cells. Cells were assessed for viability, then expression of CD3 and exclusion of B220, CD11b, CD11c, CD19, F4/80, and NK1.1 on the surface. The CD3+ T cell population was then gated to include CD4+ or CD8+ T cells. CD4+ were then gated to include those antigen-experienced (CD44+) cells producing the cytokines (**a**) IFN-γ or (**b**) IL-17A and (**c**) CD8+ were then gated to include those antigen-experienced (CD44+) cells producing the cytokine IFN-γ.

We next examined the cellular immune responses elicited by OMV immunization. Mice immunized with OMV vaccine produced significant increases in IFN-γ- and IL-17A-producing antigen-specific CD4 T cells (*p* < 0.05; *p* < 0.001 respectively), indicative of Th1 and Th17 immune responses, Figure 4a,b). Immunization with the live vaccine promoted a significant increase in IL-17A-producing antigen-specific CD4 T cells (*p* < 0.01), but did not induce a significant increase in IFN-γ-producing CD4 T cells (*p* = 0.89). Mice immunized with OMV vaccine also produced significant increases in IFN-γ-producing antigen-specific CD8 T cells (*p* < 0.05; Figure 4c). In contrast, the CD8 T cell response in mice immunized with live vaccine was not significantly different than sham-immunized mice (*p* = 0.44) (Figure 4c). These results indicate that the OMV vaccine stimulates robust helper and cytotoxic T cell responses that are superior to that induced by immunization with a live vaccine.

### 3.4. OMV Immunization Provides Protection against Glanders in Nonhuman Primates

The significant protection afforded by the OMV vaccine in the murine model of inhalational glanders led us to test its immunogenicity and efficacy in nonhuman primates. Rhesus macaques were immunized subcutaneously with OMV vaccine (*n* = 6) or saline (*n* = 4) twice, 4 weeks apart. One month later, macaques were exposed to aerosolized *B. mallei* strain China 7. The mean inhaled dose was 1.6 × 10^6^ CFU, and there was no significant difference in inhaled dose between sham-immunized and OMV-immunized animals (*p* = 0.77; Figure S3). Macaques were monitored for up to 21 days and necropsied at the study endpoint. 

**Figure 5:**
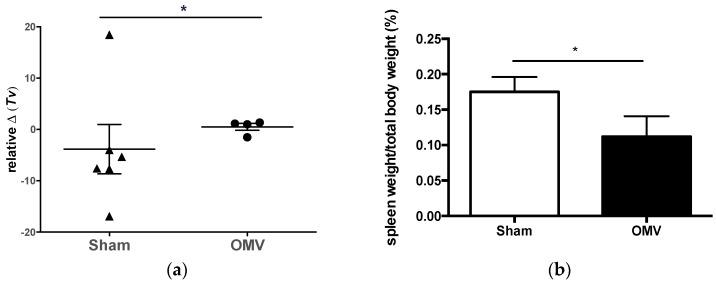
Immunization with *B. pseudomallei* OMVs provides protection against glanders disease in nonhuman primates. Pulmonary function of rhesus macaques vaccinated with OMV or saline (sham-vaccinated). (**a**) Relative change (Δ) in tidal volume (mL/breath) prior to challenge when compared to measurement +7 days after *B. mallei* aerosol challenge. Group comparison by Kolmogorov-Smirnov test, significance at * *p* < 0.05; (**b**) Spleen weight over total body weight was calculated as a percentage to quantify degree of splenomegaly (* *p* < 0.05).

All animals survived challenge with *B. mallei* over the 21 day period, despite the very high aerosol dose. Pulmonary lesions were the most common pathological finding in challenged macaques. Gross examination at necropsy revealed mild bronchopneumonia in OMV-immunized animals and more severe areas of consolidation and pleural adhesions in sham-immunized animals (Table 1). Physiological function (respiratory function) in animals prior to *B. mallei* challenge and seven days post-challenge varied according to immunization (Figure 5a). The respiratory function of OMV-immunized animals tended to be more homogenous as a group and similar to pre-challenge levels when compared to the results of the sham-immunized animals. Changes in tidal volume, which represents the volume of air breathed in upon inhalation, was significantly heterogeneous in the sham cohort when compared to OMV immunized animals (Figure 5a). Although nonsignificant, other parameters (*Mv*, *f*, EF_50_) suggest changes in respiratory function post-challenge in the sham-treated group, which may be a corollary to disease (Figure S4). Taken together, these data suggest that OMV vaccination protects against a sub-clinical pulmonary glanders infection in nonhuman primates. 

Gross examination of spleens at necropsy indicated that 2 out of 4 (50%) sham-immunized animals experienced pronounced splenomegaly, whereas all spleens appeared normal upon necropsy of OMV-immunized animals. The spleen weight to total body weight ratios were significantly higher in the sham cohort compared to that of OMV-immunized animals (*p* < 0.05) (Figure 5b). Additionally, only sham-immunized macaques harbored noticeable lesions in the spleen that, when plated for cfu, contained viable *B. mallei* (Table 1). Another striking observation was that 2 out of 4 (50%) sham-immunized animals developed cutaneous ulcers, a well-described chronic manifestation of glanders, and a plausible sign of disseminated disease (Table 1). In summary, the systemic and cutaneous pathology observed only in sham-immunized animals after *B. mallei* inhalational challenge suggests that OMV vaccination limited both the development and severity of disseminated glanders disease in macaques.

### 3.5. Immunization with OMVs Induces B. mallei Specific Antibody in Non-Human Primates 

**Figure 6:**
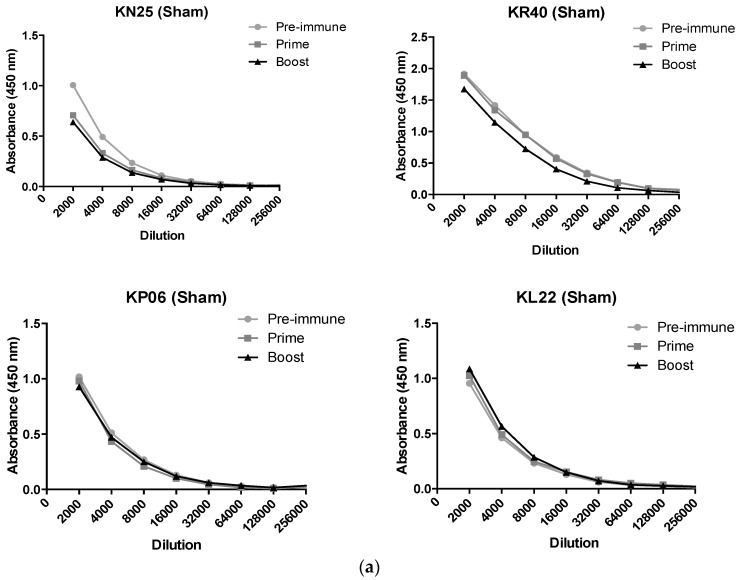

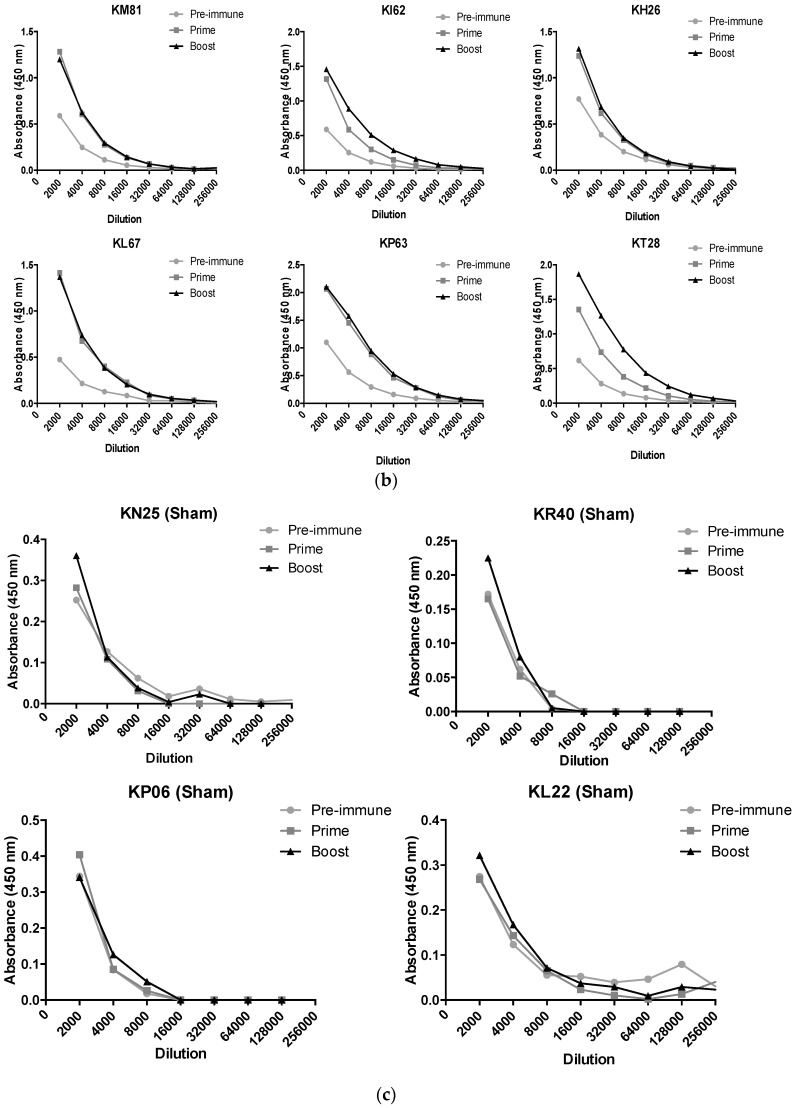

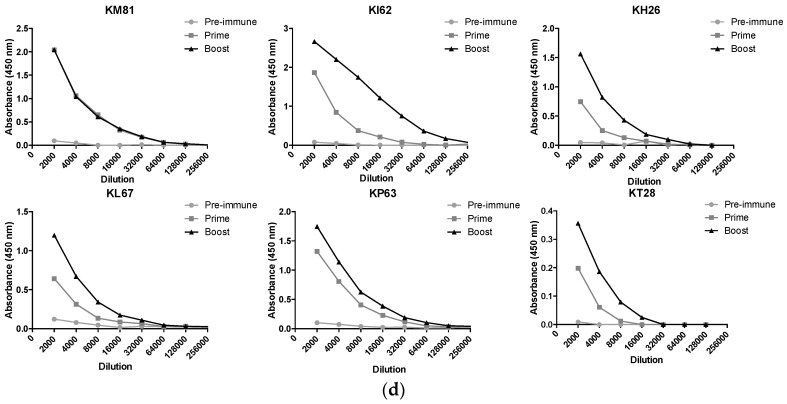
OMV immunization induces *B. mallei*-specific antibody responses in nonhuman primates. (**a**) *B. mallei*-specific and (**c**) OMV-specific IgG were measured in the sera of sham-immunized and (**b**,**d**) OMV-immunized rhesus macaques by ELISA prior to immunization (pre-immune), one month after the first dose (prime), and two weeks after the second dose (boost). Reciprocal titers are plotted against absorbance for each individual macaque.

*B. mallei*- and OMV-specific IgG titers were measured in the sera of sham-immunized and OMV-immunized rhesus macaques prior to vaccination (pre-immune), one month after the first dose (prime) and two weeks after the second dose (boost). The kinetics of the *B. mallei*- and OMV-specific antibody responses are shown individually for each animal (Figure 6). Sera from some of the animals displayed considerable immunoreactivity with *B. mallei* prior to immunization with reciprocal endpoint titers > 1:8000 (Figure 6a,b). This is not entirely unexpected because the macaques utilized in our study are raised in outdoor colonies prior to study assignment and are exposed to closely-related soil bacteria, including non-pathogenic *Burkholderia* species. Notably, all macaques assigned to our study were pre-screened at baseline by latex agglutination assay [41] (kindly provided by Paul Brett and Mary Burtnick) to confirm that they were seronegative for the pathogenic *Burkholderia*. Following OMV immunization, animals demonstrated a significant increase in *B. mallei*-specific IgG reciprocal endpoint titers (up to 1:64,000) compared to the response in sham-immunized animals (*p* < 0.01). Pre-immune titers to OMV antigens were similar in all animals (<1:4000) and increased significantly in OMV immunized macaques after both the prime (mean 1:16,000) and booster (mean 1:46,000) doses (Figure 6c,d).

Cellular immune responses were also examined by performing antigen restimulation assay on peripheral blood mononuclear cells obtained from the blood of immunized macaques two weeks after the booster dose. There was no detectable difference in antigen-specific CD4 or CD8 T cells between OMV- and sham-immunized animals.

## 4. Discussion

In this work, we demonstrate that immunization with *B. pseudomallei* OMVs provides significant cross protection against inhalational glanders in mice and nonhuman primates. Aerosol challenge of C57Bl/6 mice resulted in rapidly lethal pneumonic infection, whereas rhesus macaques resisted a very high aerosol challenge dose and developed a sub-clinical infection that persisted until the three week study endpoint. Thus, we were able to assess the protective capacity of the OMV vaccine against both the acute and chronic manifestations of glanders. OMV immunization protected mice from an otherwise lethal pneumonic glanders infection. The protection afforded by the OMV vaccine against *B. mallei* in C57Bl/6 mice is better than what we previously observed in OMV-immunized BALB/c mice challenged with *B. pseudomallei* [28]. This is likely due to the genetic differences in susceptibility to intracellular bacterial infection between C57Bl/6 and BALB/c mouse strains. Both the OMV and live attenuated strain failed to provide sterilizing immunity in mice in the current study. These results are consistent with the results of other studies evaluating *B. pseudomallei* vaccines for cross protection against glanders in mice [19,42]. The inability of *B. pseudomallei* vaccines to prevent against persistent glanders infection may be an inherent caveat of the murine model or could reflect subtle differences between protective antigenic or immunologic determinants for *B. mallei* and *B. pseudomallei* [7]. For example, differences in the number and type of ATP-binding cassette systems, such as those involved in iron acquisition, exist between *B. mallei* and *B. pseudomallei* that may influence their survival in the host [43].

Rhesus macaques were highly resistant to *B. mallei* aerosol challenge and did not develop overt pulmonary distress. This is consistent with that reported by other investigators in the field. Other nonhuman primate species, in particular African Green monkeys, are more susceptible to lethal *B. mallei* aerosol infection [44] and may be preferable for evaluation of vaccine-mediated protection against acute pneumonic glanders. However, the indolent nature of the disease in macaques observed in our study may better reflect the disease course in humans that are naturally exposed to *B. mallei* [3]. In this regard, we were able to demonstrate OMV vaccine-mediated protection against disseminated glanders infection, including the development of splenomegaly and cutaneous ulcers, in the rhesus macaque model.

Live attenuated vaccine strains often serve as the gold standard for achieving protection against intracellular bacteria, and investigational vaccine candidates should achieve equivalent or better protection in order to move forward. In this study, we present the first head-to-head comparison of the OMV vaccine platform to a live attenuated vaccine using the parent strain from which the OMVs were derived. Our results in mice convincingly demonstrated that the OMV vaccine is comparable to the live attenuated vaccine in terms of immunogenicity and protective efficacy. This is encouraging because the OMV vaccine platform is less complex and inherently safer than live attenuated vaccines. In our study, OMV immunization induced higher titers of OMV- and *B. mallei*-specific IgG than the live attenuated vaccine. This may reflect differences in surface antigen composition, vaccine uptake and recognition, or adjuvanticity and will require further studies to elucidate.

We also compared the ability of each vaccine platform to elicit cellular immune responses in mice since both humoral and cellular immunity are likely needed for complete protection [18]. We previously demonstrated the ability of the OMV vaccine to induce memory T cell responses in mice through antigen restimulation of splenocytes; however, we did not distinguish the type of cellular immunity [29]. Here, we expanded on our original work by demonstrating that OMV immunization induced a mixed Th1/Th17 CD4 T cell response in mice, as well IFN-γ-producing CD8 T cells. This totality of cellular immune responses was not achieved with the live attenuated vaccine. IFN-γ-producing T cells, like those elicited by the OMV vaccine, are especially desirable against intracellular bacteria and IFN-γ has been shown to be crucial for controlling *B. mallei* infection in mice [45]. We did not detect antigen-specific cellular immune responses in the blood of immunized macaques, likely due to their numbers being below the limit of detection using standard flow cytometric methods. It is likely that the majority of antigen specific T cells were sequestered in lymphoid tissues after immunization and were exceptionally rare in the blood [46]. 

In conclusion, *B. pseudomallei*-derived OMVs provide significant vaccine-mediated protection against both melioidosis and glanders and induce humoral and cellular immune responses. Induction of antibody, CD4 and CD8 T cells responses with an acellular vaccine formulation has traditionally proven very difficult [47], and the OMV vaccine is able to accomplish this without a requirement for exogenous adjuvant. The ability of the self-adjuvanting OMV vaccine platform to elicit both arms of the immune response explains its exceptional capacity to induce protection against both extracellular [48,49,50,51] and intracellular pathogens [28,29,52], including those capable of persistent infections. OMVs convey a number of advantages over whole cell based vaccines including oral, intranasal, and parenteral delivery options; reduced risk of vaccine-induced inflammation, and more efficient uptake and presentation by host antigen presenting cells for presentation to T cells [53]. OMV vaccines have been safely and successfully used in humans for decades to prevent Group B *Neisseria meningitidis* worldwide, and the recent approval of “Bexsero” by the Food and Drug Administration establishes precedence for OMV vaccine licensure in the United States [54]. Use of biosafety level two bacterial strains for OMV production, such as that performed here, will greatly facilitate downstream process development and manufacturing of OMV vaccines. The utility of OMV vaccines against other persistent, intracellular pathogens warrants investigation.

## Figures and Tables

**Table 1:** Pathological findings in sham- and OMV-immunized macaques infected with *B. mallei*.

Group	Animal ID	Gross Pathology		
		Lung	Spleen	Skin
**Sham**	KN25	Fibrous pleural adhesions with associated hemorrhage and focal pneumonia	Normal appearance	1.7 × 2 cm granulating ulcer present on the back
	KR40	Mild bronchopneumonia with focal areas of hemorrhage	1–2 mm white foci near capsule	None
	KP06	Marked areas of consolidation	2× enlarged	None
	KL22	Areas of consolidation suggestive of focal pneumonia	3–4× enlarged	2 cm ulcer on lower back
**OMV**	KM81	Ecchymotic hemorrhages	Normal appearance	None
	KI62	1 cm granuloma	Normal appearance	None
	KH26	Mild bronchopneumonia	Normal appearance	None
	KL67	Mild bronchopneumonia	Normal appearance	None
	KP63	Focal granulomatous pneumonia with pleuritis	Normal appearance	None
	KT28	Focal granulomatous pneumonia with pleuritis	Normal appearance	None

## Supplementary Materials

The following are available online at www.mdpi.com/2076-393X/5/4/49/s1, Figure S1: Purified Bp82 OMVs imaged by Electron Microscopy, Figure S2: OMV immunization induces IgG antibodies against numerous *B. mallei* antigens, Figure S3: *B. mallei* aerosol challenge doses for rhesus macaques immunized with sham or OMV vaccine, Figure S4: Changes in respiratory function after immunization and *B. mallei* challenge in nonhuman primates.
