# Supplementary material for: A Burkholderia pseudomallei Outer Membrane Vesicle Vaccine Provides Cross Protection against Inhalational Glanders in Mice and Non-Human Primates
Source: Vaccines (Basel). 2017 Dec 9;5(4):49. doi: 10.3390/vaccines5040049 (PMC5748615; doi:10.3390/vaccines5040049)
Supplement: Supplementary file 1 [file vaccines-05-00049-s001.pdf]

vaccines

A *Burkholderia pseudomallei* Outer Membrane Vesicle Vaccine Provides Cross Protection against Inhalational Glanders in Mice and Non-Human Primates

Sarah M. Baker, Christopher J. H. Davitt, Natalya Motyka, Nicole L. Kikendall, Kasi Russell-Lodrigue, Chad J. Roy, and Lisa A. Morici

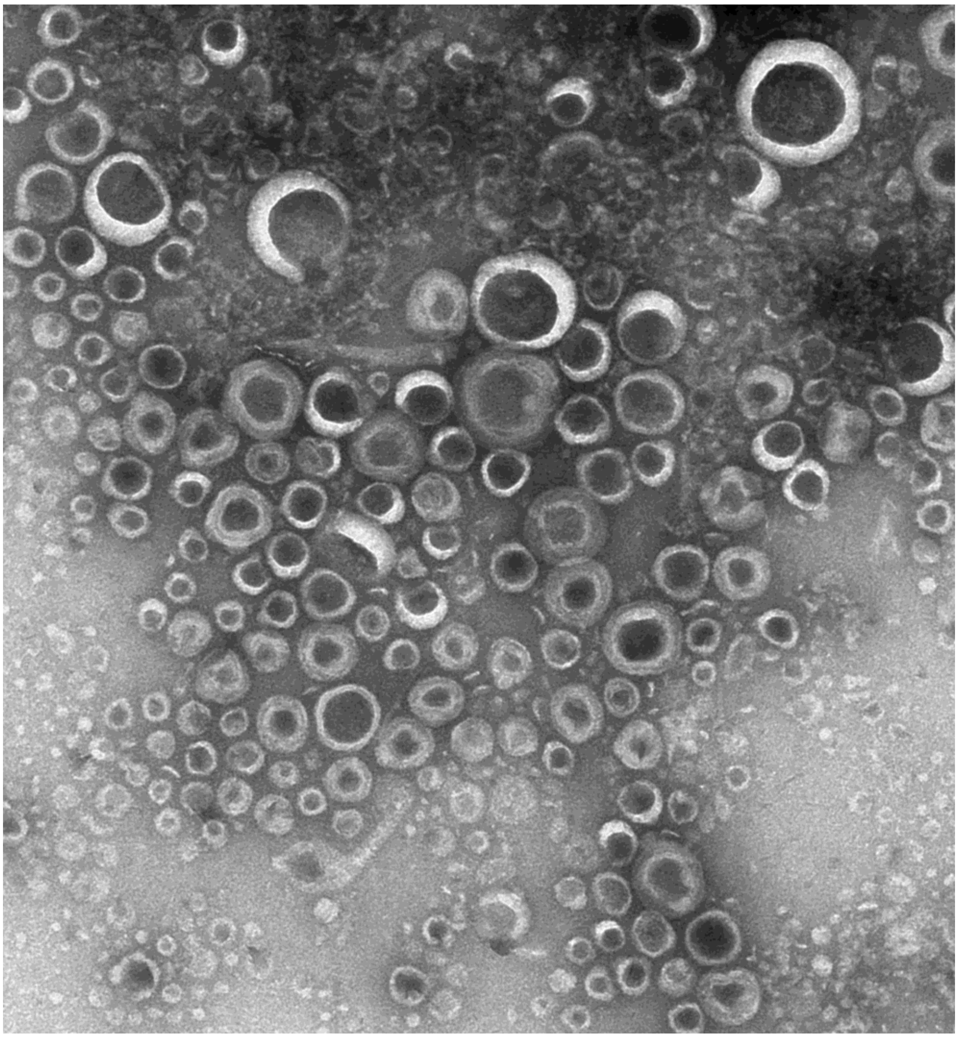

Figure S1. OMVs purified from *B. pseudomallei* strain Bp82. Purified OMVs were negatively stained with 1% uranyl acetate and imaged by transmission electron microscopy. OMVs range between 25 and 200 nm in diameter. Magnification 31,000x. Scale bar 200nm.

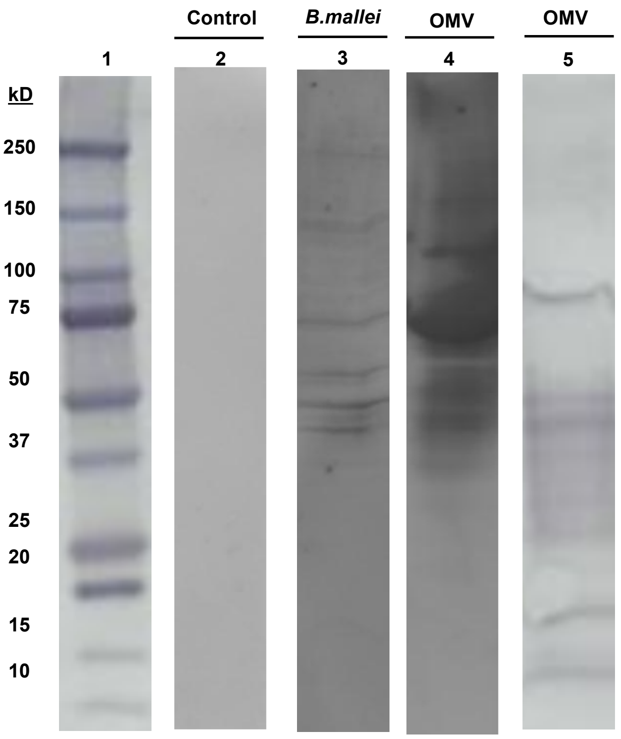

Figure S2. OMV immunization induces IgG antibodies against numerous *B. mallei* antigens. Western blot of *B. mallei* lysate (lanes 2 and 3) and OMV (lanes 4 and 5) preparations using pooled sera from sham-immunized (lane 2) or OMV immunized (lanes 3 and 4) mice (n=5 per group). OMV O-polysaccharide (OPS) in lane 5 was detected using a monoclonal antibody (Pp-Ps-W) to *B. pseudomallei* OPS to facilitate comparison of immunoreactive proteins versus OPS in *B. mallei* lysate probed with OMV immune sera. Lane 1 = molecular weight marker.

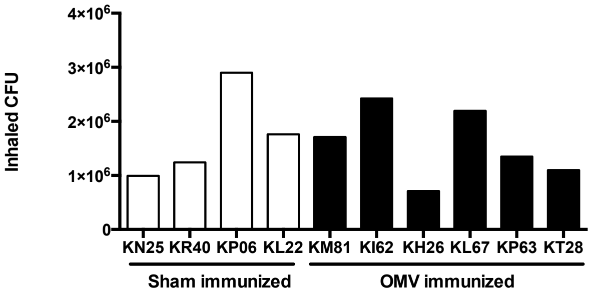

(**a**)

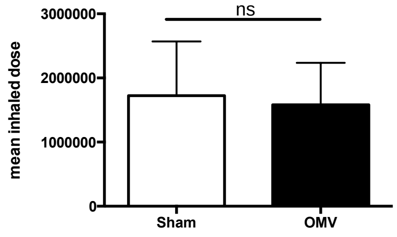

(**b**)

Figure S3. *B. mallei* aerosol challenge doses for rhesus macaques immunized with sham or OMV vaccine. (**a**) Inhaled doses of *B. mallei* were calculated for each macaque. Doses ranged from 7.1x10^5 to 2.9 x 10^6 cfu per animal. (**b**) Mean inhaled dose per immunization group. There was no difference in inhaled dose between sham immunized and OMV-immunized animals (ns = not significant, p=0.77 using students t-test).

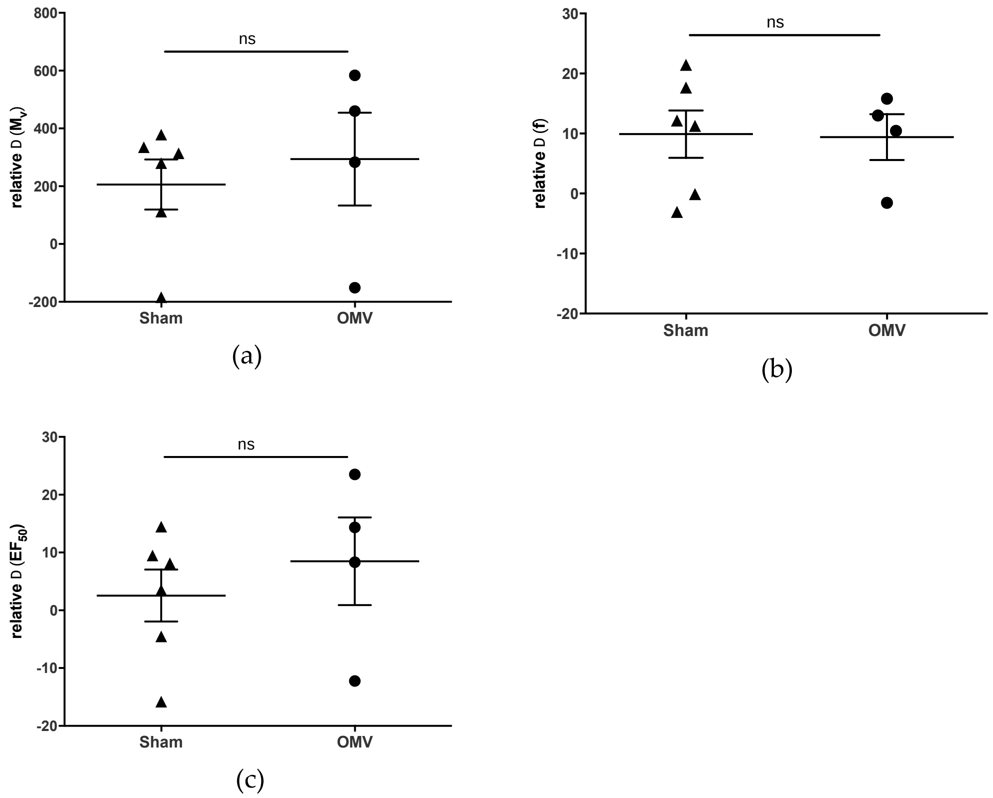

(a) (b) (c)

Figure S4. Changes in respiratory function after immunization and *B. mallei* challenge in nonhuman primates. Pulmonary function of rhesus macaques vaccinated with OMVs or saline (sham-vaccinated). Relative change (△) in each respiratory parameter prior to challenge when compared to measurement +7days after *B. mallei* aerosol challenge. (**a**) Minute volume (ml/minute); (**b**) frequency (breaths/min); (**c**) EF_{50} (ml/sec). Group comparison by Kolmogorov-Smirnov test, ns= not significant at $p<0.05$.
